# Antimicrobial resistance trends of ESKAPEE pathogens isolated from intensive care unit patients at a tertiary care hospital in Mogadishu, Somalia: a retrospective study (2020–2024)

**DOI:** 10.3389/fmicb.2026.1717344

**Published:** 2026-05-01

**Authors:** Ahmed Hassan Ibrahim, Hassan Adan Ali Adan, Mohamed Hasan Idiris, Suleiman Mohamed Asir, Said Mohamed Mohamud, Liban Abdi Nor, Yahye Sheikh Abdulle Hassan, Mustapha Goni Abatcha, Abdullahi Ahmed Tahlil, Mohamed Abdelrahman Mohamed

**Affiliations:** 1 Department of Clinical Microbiology Laboratory, Mogadishu Somali Turkey Recep Tayyip Erdoğan Training and Research Hospital, Mogadishu, Somalia; 2 Department of Emergency and Critical Care, Mogadishu Somali Turkey Recep Tayyip Erdoğan Training and Research Hospital, Mogadishu, Somalia; 3 Department of Infectious Diseases, Turkey Recep Tayyip Erdoğan Training and Research Hospital, Mogadishu, Somalia; 4 Department of Nursing and Midwifery, Faculty of Medicine and Health Sciences, Jamhuriya University of Science and Technology, Mogadishu, Somalia; 5 Division of Infectious Diseases, Department of Medicine, Escola Paulista de Medicina, Universidade Federal de São Paulo (UNIFESP), São Paulo, Brazil; 6 National Institute of Health, Ministry of Health and Human Services, Mogadishu, Somalia; 7 Faculty of Medicine and Health Sciences, Zamzam University of Science and Technology, Mogadishu, Somalia; 8 Center of Excellence for Epidemic Intelligence and Pandemic Readiness, Mogadishu, Somalia; 9 Faculty of Medicine and Health Sciences, Jamhuriya University of Science and Technology, Mogadishu, Somalia; 10 One Health Unit, Ministry of Health and Human Services, Mogadishu, Somalia; 11 Somali Microbiology Society, Mogadishu, Somalia

**Keywords:** antimicrobial resistance, ESKAPEE, hospital-acquired infection, ICU, Somalia

## Abstract

**Background:**

The ESKAPEE pathogens *Enterococcus faecium*, *Staphylococcus aureus*, *Klebsiella pneumoniae*, *Acinetobacter baumannii*, *Pseudomonas aeruginosa*, *Enterobacter* spp. and *Escherichia coli* are the leading causes of nosocomial infections and are notorious for their multidrug resistance (MDR). Data on their prevalence and resistance patterns in Somalia remain scarce. This study aims to delineate the epidemiology and antimicrobial resistance profiles of ESKAPEE isolates from the intensive care unit (ICU) patients over 5 years at Somali Turkish Recep Tayyip Erdogan Hospital, Mogadishu.

**Methods:**

A retrospective review was conducted based on microbiological data from ICU patients admitted between January 2020 and October 2024. Clinical specimens were processed using standard microbiological techniques, and isolates were identified via biochemical assays. Antimicrobial susceptibility testing was performed using the Kirby-Bauer disk diffusion method in accordance with CLSI guidelines. MDR was defined as resistance to three or more antimicrobial classes. Data were analyzed using R software v4.1.2.

**Results:**

A total of 1,632 ESKAPEE isolates were obtained from 1,632 clinical specimens. *E. coli* was the most common pathogen (42.16%), followed by *S. aureus* (17.77%), *P. aeruginosa* (17.65%), and *A. baumannii* (11.89%). The rate of MDR was higher in *A*. *baumannii* (85%) and *K*. *pneumoniae* (78%). Notably, carbapenem resistance was present in 70% of *A*. *baumannii* and 55% of *K*. *pneumoniae* isolates. There was low resistance to vancomycin among *S. aureus* isolates resistance reached approximately 6% in 2024; overall susceptibility remained 94%. Resistance to vancomycin was found in 12.5% of *E. faecium* isolates. Methicillin-resistant *S. aureus* (MRSA) increased sharply to a peak of 78.8% in 2023 before declining in 2024, highlighting unstable but concerning resistance dynamics. MDR patterns differed across pathogens, with multi-drug resistance most common among Gram-negative species.

**Conclusion:**

The high prevalence of MDR ESKAPEE pathogens, particularly carbapenem-resistant *A*. *baumannii* and *K*. *pneumoniae*, underscores the urgent need for robust antimicrobial stewardship and infection control strategies in Somali ICUs. Continuous surveillance and tailored empiric therapy protocols are critical to combating the mounting AMR threat in resource-limited settings.

## Introduction

Antimicrobial resistance (AMR) represents one of the most pressing global public health crises of the 21st century, posing a formidable challenge to modern medicine (Laxminarayan et al., 2013). The increasing prevalence of multidrug-resistant (MDR) pathogens has led to prolonged hospitalizations, escalated treatment costs, and elevated mortality rates, particularly in low-resource settings (Diop et al., 2025). According to the World Health Organization (WHO), AMR was directly responsible for 1.27 million deaths in 2019, with an additional 4.95 million deaths associated with resistant infections (Zhang et al., 2023). If current trends persist, AMR-related fatalities could reach 10 million annually by 2050, surpassing mortality from cancer and diabetes combined (Adebisi and Ogunkola, 2023).

Among the most concerning pathogens are the ESKAPEE organisms: *Enterococcus faecium, Staphylococcus aureus, Klebsiella pneumoniae, Acinetobacter baumannii, Pseudomonas aeruginosa, Enterobacter* spp., and *Escherichia coli* which are notorious for their capacity to “escape” the bactericidal effects of conventional antibiotics (Oyenuga et al., 2024). These pathogens are among the leading etiological agents responsible for hospital-acquired infections (HAIs), including bloodstream infections, pneumonia, urinary tract infections, and surgical site infections (Tacconelli et al., 2018). The WHO has classified *A. baumannii* and carbapenem-resistant *Enterobacterales* as “critical priority” pathogens. In addition, *E. faecium* (vancomycin-resistant), *S. aureus* (methicillin-resistant), and *P. aeruginosa* are designated as “high priority” due to their extensive resistance profiles and clinical impact (World Health Organization, 2024).

In sub-Saharan Africa, the burden of antimicrobial resistance is exacerbated by weak antimicrobial stewardship, inadequate diagnostic capacity, and widespread over-the-counter antibiotic use (Roca et al., 2015). Studies from the region report alarming resistance rates, including >70% resistance to third-generation cephalosporins in *K. pneumoniae* and >50% methicillin resistance in *S. aureus* (Tadesse et al., 2017). Furthermore, the lack of robust surveillance systems in many African countries hinders the accurate assessment of the crisis, obscuring its true magnitude and hindering effective policy interventions (Lubanga et al., 2024).

Somalia is a nation grappling with political instability, fragile healthcare infrastructure, and limited regulatory oversight of pharmaceuticals, faces an acute AMR crisis (Hassan et al., 2024). A recent estimate suggests that 8,400 deaths in Somalia were directly attributable to AMR in 2019, with 32,700 additional deaths associated with resistant infections (Hassan et al., 2024). Unregulated antibiotic use, frequent stockouts of essential medicines, and poor infection prevention and control (IPC) measures in hospitals further amplify the spread of resistant strains (Adam et al., 2025). Emerging data from Mogadishu have indicated high resistance rates among gram-negative bacteria, with 88.5% of *E. coli* and *K. pneumoniae* isolates resistant to fluoroquinolones, carbapenems, and third-generation cephalosporins (Omar et al., 2023). Similarly, carbapenem-resistant *A. baumannii* has been implicated in nosocomial outbreaks in intensive care units (Omar et al., 2023).

Despite these concerning trends, comprehensive data on the epidemiology of ESKAPEE pathogens in Somalia remain scarce. Most existing studies focus on specific infections (e.g., UTIs, bloodstream infections) rather than systematic AMR surveillance (Omar et al., 2023). Given the critical role of ESKAPEE pathogens in HAIs and their propensity for MDR development, understanding their resistance patterns is essential for guiding empirical therapy and infection control policies. The country’s vulnerable population may suffer from high morbidity and mortality if no action is taken urgently to combat drug-resistant bacteria.

This retrospective study aims to characterize the prevalence and antimicrobial resistance profiles of ESKAPEE pathogens isolated from clinical specimens at Recep Tayyip Erdoğan Hospital in Mogadishu, Somalia, over 5 years (2020–2024). The findings will provide critical insights into local AMR trends, informing clinical decision-making, antibiotic stewardship programs, and national AMR containment strategies in Somalia.

## Materials and methods

### Study design and setting

This retrospective observational study was conducted at the Somali Turkish Recep Tayyip Erdoğan Training and Research Hospital in Mogadishu, Somalia. The study period ran from January 1, 2020, to October 31, 2024.

### Studying population

This study included all patients admitted to the Intensive Care Unit (ICU) who had positive microbiological cultures from clinical specimens collected at least 48 h after admission, indicating nosocomial infections. The inclusion criteria included patients of any age and gender with a culture-confirmed infection. Patient data used in this study were acquired retrospectively from ICU patients.

### Sample collection and bacterial identification

Specimens, including blood, urine, cerebrospinal fluid, respiratory secretions, pleural and peritoneal fluids, and wound swabs, were obtained aseptically. Cultures were performed on blood agar, eosin methylene blue (EMB) agar, and chocolate agar, followed by incubation under appropriate conditions. Bacterial isolates were initially identified based on colony morphology and Gram staining.

For Gram-positive isolates, further identification was conducted using standard biochemical tests. Catalase and coagulase tests were performed for the identification of *S. aureus*, while bile esculin hydrolysis and NaCl tolerance testing were used for the identification of *Enterococcus* spp. Gram-negative isolates were identified using biochemical testing with the BBL Crystal enteric/non-fermenting identification system (Becton Dickinson). Confirmatory identification was performed using the BBL Crystal AutoReader.

### Antimicrobial susceptibility testing

Susceptibility profiles were determined using the Kirby-Bauer disk diffusion method on Mueller-Hinton agar, in accordance with the guidelines of the Clinical and Laboratory Standards Institute (Hindler and Munro, 2024). Bacterial suspensions were standardized to 0.5 McFarland turbidity before inoculation. Antibiotic disks included agents relevant to local treatment protocols and global standards. The antibiotics tested were representative of several classes, including carbapenems (meropenem-MPM 10 μg), fluoroquinolones (levofloxacin-LEV 5 μg, ciprofloxacin-CIP 5 μg), cephalosporins (ceftazidime-CAZ, cefixime-CFM, cefepime-FEP, ceftriaxone-CRO), penicillins with β-lactamase inhibitors (piperacillin/tazobactam-TPZ, amoxicillin/clavulanic acid-AMC), aminoglycosides (amikacin-AMK, gentamicin-GN), sulfonamides (trimethoprim/sulfamethoxazole-SXT 10 μg), tetracyclines (tetracycline-TET 30 μg), glycopeptides (teicoplanin-TEC 30 μg, vancomycin-VA 30 μg), beta-lactams (penicillin-P 10 units, piperacillin-PIP 100 μg), macrolides (erythromycin-E 30 μg), streptogramins (quinupristin-dalfopristin-QD15 μg), lincosamides (clindamycin-DA 2 μg), oxazolidinones (linezolid-LNZ30 μg), polymyxins (colistin-CT 10 μg), nitrofurans (nitrofurantoin-NITR10 μg), and monobactams(aztreonam-ATM 10 μg). Notably, aminoglycosides, carbapenems, and fluoroquinolones were tested exclusively against Gram-negative isolates, while glycopeptides, oxazolidinones, and lipopeptides were tested only against Gram-positive organisms. Accordingly, agents such as amikacin were not tested against *E. faecium*. Quality control procedures were conducted using standard American Type Culture Collection (ATCC) reference strains, including *E. coli* ATCC 25922, *S. aureus* ATCC 25923, *P. aeruginosa* ATCC 27853, and *E. faecium* ATCC 29212. Cefoxitin disk diffusion was performed for *S. aureus* to screen for methicillin resistance in accordance with CLSI guidelines. Multidrug resistance (MDR) was defined as resistance to at least one agent in three or more antimicrobial classes.

### Data analysis

Data were entered and analyzed using R software version 4.1.2. Descriptive statistics summarized pathogen prevalence and resistance rates. Categorical variables were expressed as frequencies and percentages; continuous variables as means ± standard deviations or medians with interquartile ranges. Associations between variables were tested using the Chi-square or Fisher’s exact tests, with significance set at *p*<0.05. The χ^2^-test evaluated associations between MDR status and demographic variables, including age group, sex, ICU ward, specimen type, and organism species.

### Ethical considerations

As a retrospective study using de-identified laboratory data, ethical approval was granted by the hospital’s Institutional Review Board (Ref number: MSTH/17212), with a waiver of informed consent.

## Results

### Patient demographics and specimen distribution

During the period of 5 years (2020–2024), the descriptive characteristics of the total sample of 1,632 clinical specimens collected from patients in the ICU unit of the Recep Tayyip Erdoğan Hospital in Mogadishu, Somalia, and tested positive for ESKAPEE pathogens are presented in Table 1. Various age groups comprised the patient population. Children (0–17 years of age) made up the largest group at 30.94%, followed by middle-aged adults (40–64 years of age), at 27.02%, and young adults (18–39 years of age), at 23.41%. 18.63% of the patients were elderly patients aged 65 or older. About 37.5% of participants were males, and 62.5% were females. Samples were most frequently collected from the Emergency ICU (43.32%) and the General ICU (26.29%) units. Most of the clinical samples were primarily from blood (42.46%) and other fluids (29.60%). The highest number of specimens was recorded in 2022, accounting for 24.14% of the total samples (Table 1).

**TABLE 1:** Descriptive characteristics of the total patient sample.

Characteristic	Category	Frequency (N)	Percentage (%)
Age	Child (0–17)	505	30.94
	Young adult (18–39)	382	23.41
	Middle-aged (40–64)	441	27.02
	Elderly (65+)	304	18.63
Gender	Female	612	37.50
	Male	1,020	62.50
Department	Coronary ICU	41	2.51
	Emergency ICU	707	43.32
	General ICU	429	26.29
	Neonatal ICU	346	21.20
	Pediatric ICU	109	6.68
Organism	*Acinetobacter baumannii*	194	11.9
	*Enterobacter spp.*	25	1.5
	*Enterococcus faecium*	28	1.7
	*Escherichia coli*	663	40.6
	*Klebsiella pneumoniae*	144	8.8
	*Pseudomonas aeruginosa*	288	17.7
	*Staphylococcus aureus*	290	17.8
Sample group	Blood	693	42.46
	Catheter	16	0.98
	Respiratory/ENT	440	26.96
	Other fluids	483	29.60
Year	2020	304	18.63
	2021	339	20.77
	2022	394	24.14
	2023	309	18.93
	2024	286	17.52
Total		1,632	100.00

### Prevalence of pathogens including *Escherichia coli*

Among the 1,632 isolates analyzed, *E. coli* was the most frequently identified pathogen (688/1,632; 42.16%), followed by *S. aureus* (290; 17.77%), *P. aeruginosa* (288; 17.65%), *A. baumannii* (194; 11.89%), *K. pneumoniae* (144; 8.82%), and *E. faecium* (28; 1.72%) (Table 1).

**FIGURE 1:**
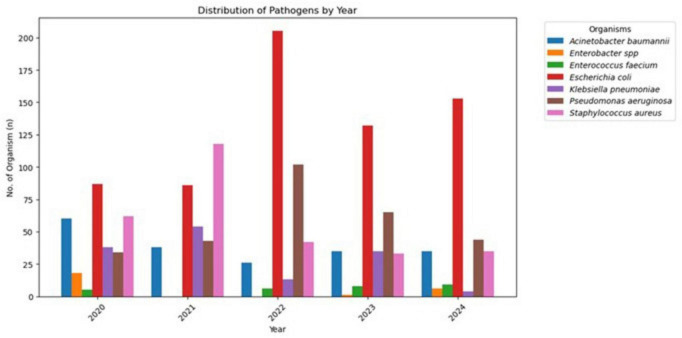
Distribution of ESKAPEE pathogens by year (2020–2024).

As shown in Figure 1, the annual isolation frequency of these strains has remained relatively stable over the past 5 years. Throughout all years, *E. coli* has consistently been the most frequently isolated organism, reaching a peak in 2022. While *A. baumannii*, *P. aeruginosa*, and *S. aureus* maintained a high presence at regular intervals, *S. aureus* cases increased in 2021, and *P. aeruginosa* experienced a rise in 2022. Despite stable levels, *K. pneumoniae* and *E. faecium* continue to pose significant threats (Figure 1).

**FIGURE 2:**
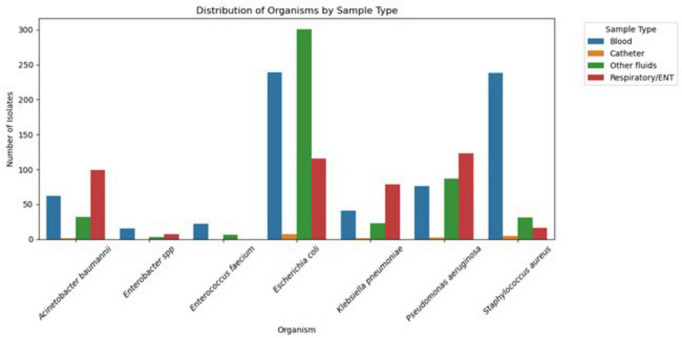
Distribution of ESKAPEE pathogens by specimen type (2020–2024).

The distribution of ESKAPEE isolates based on clinical specimens collected from patients is shown in Figure 2. All ESKAPEE strains were mainly isolated from blood samples at 693/1,632 (42.46%)., followed by other clinical fluid specimens. *S. aureus*, *K. pneumoniae*, *A*. *baumannii*, *P*. *aeruginosa*, and *E*. *coli* were recovered from respiratory/ear, nose and throat (ENT) samples. In contrast, only *S*. *aureus* and *E*. *coli* were recovered from catheter samples *at* 34, 20%, respectively.

### Antimicrobial resistance patterns of ESKAPEE pathogens

The resistance patterns for the ESKAPEE pathogens were tested for several antibiotic classes, notably carbapenems, fluoroquinolones, cephalosporins, penicillins and beta-lactamase inhibitors, aminoglycosides, sulfonamides, tetracyclines, glycopeptides, lincosamides, beta-lactams, macrolides, streptogramins, oxazolidinones, polymyxins, nitrofurans, and monobactams. The annual analysis of antibiotic resistance trends was evaluated for all pathogens to understand resistance patterns over 5 years using a line chart and tables.

The analysis of antibiotic resistance trends for *E. faecium* from 2020 to 2024 revealed a significant increase in resistance to several key antibiotics (Table 2). Among the seven antibiotics tested, resistance to penicillins and tetracycline surged dramatically, reaching 100% by 2024, while erythromycin resistance increased sharply to nearly 90%. Trimethoprim/sulfamethoxazole has shown fluctuating but ultimately high levels of resistance. Critically, the emergence and rise of vancomycin resistance to approximately 11–12.5% by 2023–2024 presents a major therapeutic challenge. In contrast, linezolid, and nitrofurantoin consistently demonstrated excellent activity, remaining highly effective against *E. faecium* isolates throughout the surveillance period. Linezolid maintained susceptibility rates exceeding 95%.in this study. Overall, despite missing data in certain years, the results indicate a marked increase in multidrug resistance in *Enterococcus faecium*, with limited remaining effective treatment options. It should be noted that no *Enterococcus faecium* isolates were reported in 2021; therefore, resistance rates were not calculated for that year and should not be interpreted as zero resistance.

**TABLE 2:** *Enterococcus faecium* antibiotic resistance trend (2020–2024).

Year	P R (%)	E R (%)	VA R (%)	TET R (%)	SXT R (%)	LNZ R (%)	NITR R (%)
2020	_	0 (0.0%)	0 (0.0%)	_	3 (60.0%)	0 (0.0%)	0 (0.0%)
2021	_	_	_	_	_	_	_
2022	3 (50.0%)	3 (50.0%)	0 (0.0%)	_	1 (16.7%)	0 (0.0%)	0 (0.0%)
2023	4 (50.0%)	7 (87.5%)	1 (12.5%)	2 (25.0%)	3 (37.5%)	0 (0.0%)	0 (0.0%)
2024	9 (100.0%)	8 (88.9%)	1 (11.1%)	9 (100.0%)	6 (66.7%)	1 (11.1%)	1 (11.1%)

*P, penicillin; E, erythromycin; VA, vancomycin; TET, tetracycline; SXT, sulfamethoxazole/trimetoprim; LNZ, linezolid; NITR, Nitrofurantoin. **R is the no. of resistance. ***“_” No isolates are tested.

**FIGURE 3:**
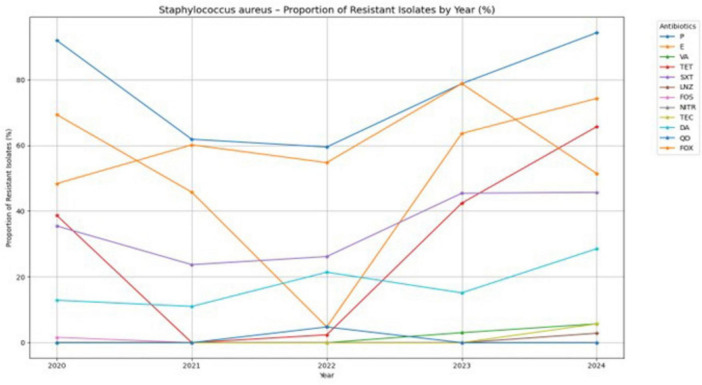
Antibiotic resistance trends of *Staphylococcus aureus* (2020–2024).

In Figure 3, the antibiotic resistance trends for *S. aureus* are shown. The resistance to penicillins has remained extremely high, at nearly 95%, while erythromycin resistance also remained significant until 2024, at approximately 74%. After an initial decrease, resistance to tetracyclines surged back to around 65%. Clindamycin and trimethoprim/sulfamethoxazole exhibited moderate and gradually increasing resistance, but the most concerning finding was the noticeable emergence of vancomycin resistance in 2024, reaching nearly 6%. This potential increase in vancomycin-intermediate and vancomycin-resistant *S. aureus* (VISA/VRSA) bacteria is a serious public health issue that demands urgent attention. However, on a brighter note, medications such as teicoplanin, quinupristin/dalfopristin, linezolid, fosfomycin, and nitrofurantoin largely retained their effectiveness, with minimal resistance throughout the study period.

**FIGURE 4:**
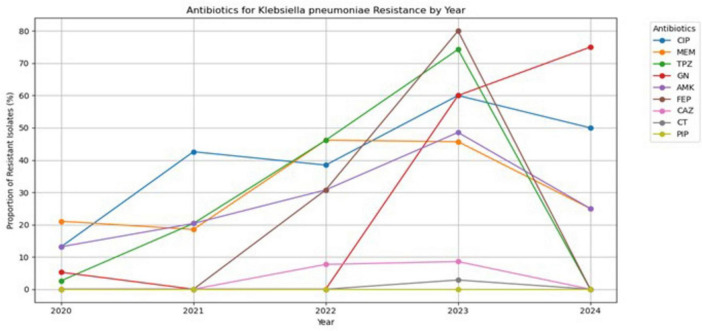
Antibiotic resistance trends of *Klebsiella pneumoniae* (2020–2024).

Figure 4 illustrates the annual trend in antibiotic resistance found in *K. pneumoniae* isolates from patients. Among the nine antibiotics tested against *K. pneumoniae*, cefepime had the highest resistance rate of 90%, reaching a peak in 2023, followed by gentamicin and piperacillin/tazobactam, with resistance rates of nearly 80%. The development of resistance to cefepime, a commonly used fourth-generation cephalosporin antibiotic, is problematic when the infection is caused by carbapenem-resistant *K. pneumoniae* and can lead to treatment failure. Likewise, resistance to ciprofloxacin, amikacin, and meropenem has been relatively high over the years. Moreover, meropenem resistance fluctuates over time; this antibiotic is only used in severe or MDR infections as a last resort. colistin, in contrast, did not have a relative resistance rate of more than 10% to *K. pneumoniae* over the study period.

**FIGURE 5:**
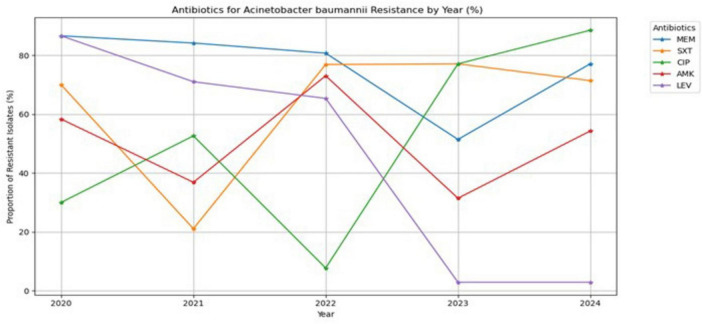
Antibiotic resistance trends of *Acinetobacter baumannii* (2020–2024).

Figure 5 shows an analysis of antibiotic resistance trends in *A. baumannii* from patients admitted to the hospital’s ICU between 2020 and 2024, revealing a complex and changing pattern across five antibiotics tested. The two antibiotics, meropenem and trimethoprim-sulfamethoxazole, consistently show very high resistance rates, often surpassing 70–80% throughout the period observed. These critical antibiotics face a significant challenge, reducing their clinical effectiveness. In stark contrast, levofloxacin resistance showed a remarkable and sustained decrease from about 65% in 2022 to < 5% in 2023 and 2024, highlighting a rare and highly significant positive development. The marked reduction in levofloxacin resistance observed in *A. baumannii* between 2022 and 2024 may reflect changes in antibiotic prescribing practices within the ICU, particularly reduced fluoroquinolone use following antimicrobial stewardship interventions. Additionally, the small number of isolates in later years may have influenced the observed resistance rates. Further prospective surveillance and molecular studies are needed to determine whether this trend represents a true epidemiological shift. Ciprofloxacin and amikacin exhibited dynamic and fluctuating resistance patterns, marked by sharp declines and subsequent rebounds, indicating a more responsive epidemiological landscape but still presenting substantial challenges to maintaining consistent therapeutic effectiveness. These resistance trends suggest limited treatment options for *A. baumannii* infections, leading to increased morbidity, mortality, and healthcare costs. Most significantly, the overall pattern of resistance trends for *A. baumannii* is characterized by considerable complexity and heterogeneity across numerous antibiotic classes. The data show that while some agents face consistently high levels of resistance, others have more dynamic fluctuations or even downward trends.

**FIGURE 6:**
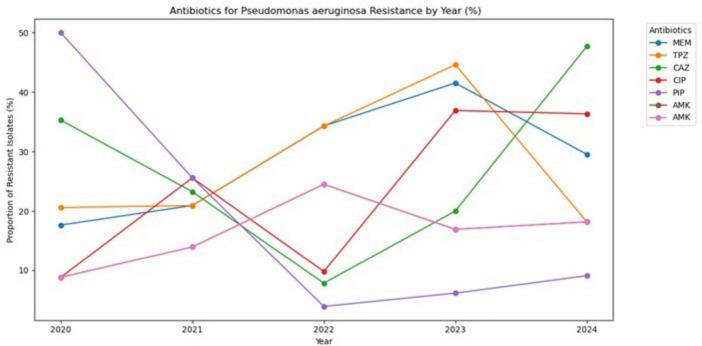
Antibiotic resistance trends of *Pseudomonas aeruginosa* (2020–2024).

The provided chart (Figure 6) shows trends in antibiotic resistance for *P. aeruginosa* against seven different antibiotics. Data reveal highly dynamic and variable patterns across all drugs, with some medications experiencing dramatic fluctuations and others showing gradual changes. Resistance levels to the meropenem and piperacillin-tazobactam showed significant fluctuations, peaking at over 40% in 2023 before declining in 2024; however, carbapenem resistance remains a serious concern. Ceftazidime and ciprofloxacin exhibited a troubling “V-shaped” trend, with resistance decreasing in 2022 only to sharply rise again, with ceftazidime’s resistance nearing 50% in 2024. Although susceptibility to piperacillin-tazobactam improved during the early years of the study, a modest increase in resistance was observed in the final year. Amikacin resistance remained moderate but showed notable year-to-year variability, highlighting the heterogeneous susceptibility patterns of *P. aeruginosa* and emphasizing the importance of continuous local surveillance.

**FIGURE 7:**
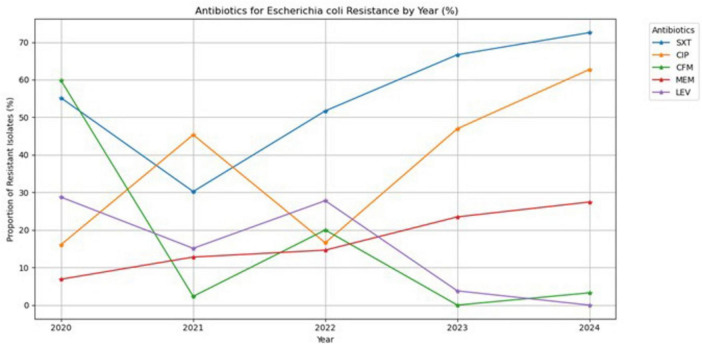
Antibiotic resistance trends of *Escherichia coli* (2020–2024).

Figure 7 shows the percentage of *E. coli* resistant to five antibiotics over 5 years, from 2020 to 2024. Resistance to trimethoprim/sulfamethoxazole generally increased over the study period, starting at about 55% in 2020 and rising sharply to over 70% in 2024. Ciprofloxacin resistance demonstrated a rapid increase, rising from approximately 40% in 2021 to nearly 65% in 2024. Notably, resistance to cefixime and levofloxacin has remained low or decreased over recent years. Resistance to meropenem has steadily and moderately increased.

Table 3 illustrates the trends in antibiotic resistance among *Enterobacter spp.* isolates during the study period. antimicrobial resistance demonstrated a predominantly increasing trend over the study period, although interpretation is limited by the absence of isolates in 2021 and 2022. Resistance to sulfamethoxazole/trimethoprim increased from 38.9% in 2020 to 66.7% in 2024, while ciprofloxacin resistance rose markedly from 11.1 to 50.0%. Similarly, resistance to meropenem and gentamicin showed an upward trend, increasing from 22.2 to 33.3% and from 11.1 to 33.3%, respectively, indicating a concerning decline in antibiotic effectiveness. Amikacin exhibited an increasing resistance trend from 5.6% in 2020 to 33.3% in 2024, although it remained relatively more effective compared to other agents. A consistently high resistance level was observed for amoxicillin/clavulanic acid in 2020 (77.8%); however, this antibiotic was not tested in 2023 and 2024, limiting further trend analysis. Notably, a peak resistance of 100% across all tested antibiotics in 2023 represents an apparent extreme increase but should be interpreted cautiously due to the very small sample size. Overall, despite data gaps, the findings suggest a progressive increase in antimicrobial resistance in *Enterobacter* spp.

**TABLE 3:** *Enterobacter* spp. antibiotic resistance trend (2020–2024).

Year	SXT R (%)	CIP R (%)	MEM R (%)	GN R (%)	AMC R (%)	AMK R (%)
2020	7 (38.9%)	2 (11.1%)	4 (22.2%)	2 (11.1%)	14(77.8%)	1 (5.6%)
2021	_	_	_	_	_	_
2022	_	_	_	_	_	_
2023	1 (100.0%)	1 (100.0%)	1 (100.0%)	1 (100.0%)	_	_
2024	4 (66.7%)	3 (50.0%)	2 (33.3%)	2 (33.3%)	_	2 (33.3%)

*“_”No isolates in 2021 and 2022. *“_” Not tested. *SXT, sulfamethoxazole/trimethoprim; CIP, ciprofloxacin; MEM, meropenem; GN, gentamicin; AMC, amoxillin/clavulanic acid; AMK, amikacin.

**FIGURE 8:**
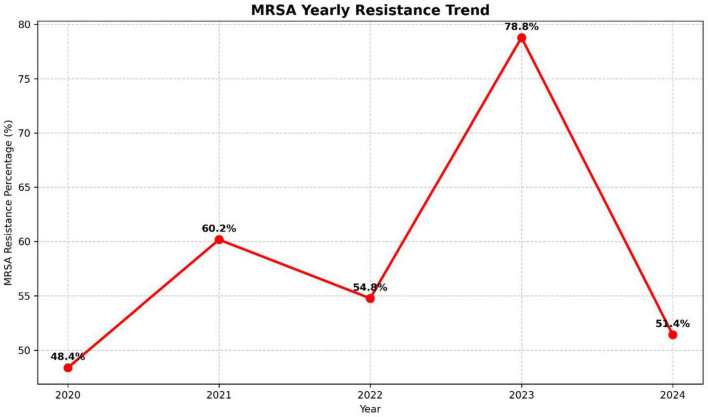
MRSA prevalence (2020–2024).

Figure 8 demonstrates the annual trend of methicillin-resistant *S. aureus* (MRSA). The proportion of MRSA isolates increased from 48.4% in 2020 to 60.2% in 2021, followed by a slight decline to 54.8% in 2022. A sharp surge was observed in 2023, with MRSA prevalence peaking at 78.8%, before declining again to 51.4% in 2024.

### Factors associated with multidrug resistance

As shown in Table 4, the incidence of multidrug resistance (MDR) increased directly with age, starting at 41.2% in children (0–17 years old) and rising to a peak of 56.1% in elderly patients (65 years and older) (χ^2^ = 21.01, df = 3, *p* < 0.001). Similarly, the patient’s admitted department was significantly associated with MDR (χ^2^ = 21.08, df = 4, *p* < 0.001), with the Coronary ICU showing a higher proportion of MDR cases, where nearly two-thirds were (65.9%). The neonatal ICU had the lowest rate at 39.2%. A correlation exists between the type of organism and MDR; *A. baumannii* (77.3% MDR) and *E. faecium* (75.0% MDR) were significantly more likely to be resistant. In contrast, *P. aeruginosa* had a much lower MDR rate of 31.5% (χ^2^ = 108.55, df = 5, *p* < 0.001). A few characteristics were not significantly related to MDR (*p* > 0.05), such as gender, where both females and males had similar proportions of MDR: 50.7 and 47%, respectively. Similarly, sample type also showed no significant relationship to MDR (*p* > 0.05). The proportion of MDR across all clinical sample types was similar, ranging from 46.0 to 53.2% in Blood and other fluids.

**TABLE 4:** Association between patient and sample characteristics and multidrug resistance (MDR).

Characteristic	Category	MDR		χ^2^(df)	*P*-value
		No n (%)	Yes n (%)		
Age category	Child (0–17)	295 (58.8)	207 (41.2)	**21.01 (3)**	**<0.001**
	Young adult (18–39)	203 (53.1)	179 (46.9)		
	Middle-aged (40–64)	209 (47.4)	232 (52.6)		
	Elderly (65+)	133 (43.9)	170 (56.1)		
Department	Coronary ICU	14 (34.1)	27 (65.9)	**21.08 (4)**	**<0.001**
	Emergency ICU	353 (50.0)	353 (50.0)		
	General ICU	203 (47.4)	225 (52.6)		
	Neonatal ICU	209 (60.8)	135 (39.2)		
	Pediatric ICU	61 (56.0)	48 (44.0)		
Gender	Female	302 (49.3)	310 (50.7)	**1.99 (1)**	**0.158**
	Male	538 (53.0)	478 (47.0)		
Sample type	Blood	373 (54.0)	318 (46.0)	**6.50 (3)**	**0.090**
	Catheter	8 (50.0)	8 (50.0)		
	Respiratory/ENT	233 (53.2)	205 (46.8)		
	Other fluids	226 (46.8)	257 (53.2)		
Organism name	*Acinetobacter baumannii*	44 (22.7%)	150 (77.3%)	**110.42 (6)**	**<0.001**
	*Enterobacter spp.*	17 (68.0%)	8 (32.0%)		
	*Enterococcus faecium*	7 (25.0%)	21 (75.0%)		
	*Escherichia coli*	358 (54.1%)	304 (45.9%)		
	*Klebsiella pneumoniae*	74 (51.4%)	70 (48.6%)		
	*Pseudomonas aeruginosa*	196 (68.5%)	90 (31.5%)		
	*Staphylococcus aureus*	144 (49.8%)	145 (50.2%)		

Bold values indicate statistically significant associations (*p* < 0.05).

## Discussion

In this study, *E. coli*, *S. aureus*, and *P. aeruginosa* were the most frequently isolated pathogens from ICU patients. Pathogens in the ESKAPEE group have been linked to nosocomial infections, hospitalizations, and MDR infections throughout the world, particularly in low- and middle-income countries (Abukhalil et al., 2024). Studies have shown that the burden of ESKAPEE pathogens causing nosocomial infections is 2–3 times higher in low- and middle-income countries (LMICs) than in high-income countries (Ayobami et al., 2022). Most health experts agree that the high prevalence of this disease in LMICs is primarily due to pre-existing challenges in the health care system, limited resources for infection prevention and control, coupled with the high levels of antimicrobial resistance (Ali et al., 2024). This 5-year retrospective analysis highlights the substantial burden of the ESKAPEE pathogen and the antibiotic susceptibility in the ICU setting of the Mogadishu Somali-Turkey Recep Tayyip Erdogan Hospital. There is a paucity of such data, and this is the first time such data have been published in Somalia. To fill the knowledge gap, this will enhance infection control, prevention, and stewardship; therefore, it is essential to understand the levels of these pathogens. In this study, 30.94% of the samples were collected from children and 27.02% from middle-aged adults. Moreover, the vast majority of samples were collected from the Emergency ICU and the General ICU units. An increased risk of ESKAPEE infection in children and middle-aged adults, particularly in intensive care units, has been linked to several factors associated with host susceptibility factors, such as immunological status, comorbidities, healthcare exposures, such as devices and antibiotics, and nosocomial transmission risk (Bereanu et al., 2024).

In this investigation, the most frequently isolated bacteria from hospitals’ ICU units were *E. coli*, *S. aureus*, and *P. aeruginosa*. *A. baumannii*, *K. pneumoniae*, and *E. faecium* were all found in low numbers. ESKAPEE bacteria have been reported as being responsible for hospital-related infections in Romania (Arbune et al., 2021), Gabon (Mouanga-Ndzime et al., 2023), Zambia (Namukonda et al., 2025), and South Africa (Mthombeni et al., 2024). The prevalence of gram-negative bacteria was high, particularly *E. coli*, suggesting a substantial burden of urinary tract, gastrointestinal, and nosocomial infections (Mouanga-Ndzime et al., 2023). In most cases, UTIs are caused by gram-negative bacteria, such as *E. coli*. However, given the presence of *S. aureus*, gram-positive bacteria should also be addressed because they are often associated with soft tissue or skin infections. UTIs are most often caused by gram-negative bacteria, particularly *E. coli* and *K. pneumoniae*, followed by *Proteus mirabilis*), with *E. faecium* as main Gram-positive uropathogen. In another study by Mouanga-Ndzime et al. (2023), the main ESKAPEE detected in UTIs were 35% *E. coli*, 34% *K. pneumoniae*, 8% *E. faecium*, and *S. aureus* accounted for 6%. However, detecting bacteria in the samples does not necessarily indicate infection; it may instead suggest colonization (Mouanga-Ndzime et al., 2023). The data in this study provide valuable information about the local epidemiology of circulating bacteria in healthcare facilities.

The emergence of AMR is becoming increasingly concerning worldwide because it is not limited to developing countries alone. Due to globalization and commercialization, humans and goods are transported across the globe. Both developed and developing nations are experiencing a rising incidence of AMR among ESKAPEE pathogens (Oliveira et al., 2024). *E. faecium* demonstrated higher resistance to penicillins, tetracycline, and erythromycin, whereas resistance to vancomycin remained low. Susceptibility to linezolid remained very high throughout the study period. *E. faecium* at the University Hospital of Palermo, Italy, was resistant to ampicillin, vancomycin, teicoplanin, gentamicin, linezolid, and imipenem (Huang et al., 2025). It is very alarming that *E. faecium* has become resistant to the antibiotics that are commonly used in clinical infections. Many strains of *E. faecium* are resistant to multiple antibiotics, including ampicillin, vancomycin, and gentamicin (Arias and Murray, 2012). WHO has classified vancomycin-resistant *E. faecium* as a critical pathogen because there are limited treatment options, posing a serious threat to public health (Almeida-Santos et al., 2025). Approximately 75.0% of the *E. faecium* were resistant to multiple antibiotic types, making it difficult to treat them (Turner et al., 2024).

It was observed that *S. aureus* was highly resistant to penicillin and erythromycin, followed by tetracycline. There was a moderate and gradually increasing resistance to clindamycin and trimethoprim/sulfamethoxazole. However, resistance to vancomycin was minimal. This potential occurrence of vancomycin-resistant *S. aureus* bacteria is a serious public health issue that demands urgent attention. even low-level resistance is clinically significant because vancomycin is a last-line agent for MRSA.

While most *S. aureus* isolates approximately 94% are susceptible to vancomycin some strains have developed reduced susceptibility or complete resistance (Tawfeek et al., 2024). Vancomycin-resistant *S. aureus* (VRSA) infections are challenging to treat but remain rare. While methicillin-resistant *S. aureus* (MRSA) is commonly reported in East African countries, vancomycin-resistant *S. aureus* (VRSA) remains rare and was not systematically assessed in the present study (Belete et al., 2023). Additionally, an increase in methicillin-resistant *S. aureus* may contribute to the emergence of VRSA (Tarai et al., 2013). Studies have indicated that in Ethiopia, the prevalence of VRSA varies, emphasizing the need for strong diagnostics and surveillance (Belete et al., 2023). The different rates of VRSA can be linked to factors such as poor sanitation, limited diagnostics, and excessive antibiotic use.

The increasing prevalence of antimicrobial resistance among gram-negative bacteria in healthcare settings is a major public health concern. These organisms exhibit both intrinsic and acquired resistance mediated through multiple complementary mechanisms. Reduced outer membrane permeability limits antibiotic entry, while enzymatic degradation particularly through the production of β-lactamases and carbapenemases directly inactivates antimicrobial agents. In addition, overexpression of efflux pump systems reduces intracellular drug concentrations, and alterations in antibiotic target sites further diminish drug efficacy (Parajuli et al., 2017). Acquired resistance is often facilitated by horizontal gene transfer via plasmids, integrons, and gene cassettes, enabling rapid dissemination of resistance determinants within and between bacterial species (Vivekanandan et al., 2025).

In this study, the occurrence of AMR across *K. pneumoniae*, *Acinetobacter baumannii*, *P. aeruginosa*, and *E. coli* appears to be a concern due to its negative impact on patients, including difficulty in treatment, hospitalization, and increased healthcare-related financial costs; the WHO is identifying them as a priority bacterial pathogens.

Cefepime resistance in *K. pneumoniae* peaked in 2023 at 75%, followed by gentamicin and piperacillin/tazobactam, both with resistance levels exceeding 80%. *K. pneumoniae* resistance to fourth-generation cephalosporins is a serious health concern. Cephalosporin drugs are commonly used for treating bloodstream infections and pneumonia. Resistance to cephalosporins is primarily mediated by the production of β-lactamase enzymes, including extended-spectrum β-lactamases (ESBLs) and AmpC β-lactamases, which hydrolyze cephalosporins and render them ineffective. In addition to enzymatic degradation, resistance may also involve reduced outer membrane permeability, overexpression of efflux pumps, and alterations in penicillin-binding proteins that decrease antibiotic binding affinity (Pipitò et al., 2025). According to a retrospective cross-sectional study of hospitalized patients in Palestine, there was high resistance to extended-spectrum cephalosporins, including 93.5% ceftazidime, 94.9% ceftriaxone, and 93.4% cefotaxime (Abukhalil et al., 2024).

*A. baumannii* has consistently displayed a higher resistance rate to both meropenem and trimethoprim-sulfamethoxazole, often exceeding 70–80%. The resistance of *A. baumannii* to meropenem is known as meropenem -resistant *A. baumannii*, and it is a serious concern because it often leads to lung, blood, and UTI infections, especially in immunocompromised patients. In another study, it was also found that *A. baumannii* was resistant to carbapenem (meropenem, which represented 98% of the resistance), imipenem (which represented 100%), and cephalosporins (96–98%) (Al-Rashed et al., 2023). Moreover, *A. baumannii* poses a significant threat in East African countries due to its increased resistance to several antibiotics, including carbapenems. Resistance mechanisms include the production of carbapenemases such as NDM-1 and OXA-23, as well as the loss of outer membrane proteins (Agyepong et al., 2023).

Three carbapenem resistance gene structures were identified: *blaOXA-23, blaOXA-51-like*, and *blaNDM-1* (Joshi et al., 2017). It should be noted that molecular detection of carbapenem resistance genes was not performed in this study; the discussion of resistance genes is based on previously published regional evidence. Among all, *blaOXA-23* was the primary carbapenem-resistance mechanism identified, as reported in other African countries. Limited surveillance data exist for the entire East Africa region, which has reported an increase in cases of MDR and carbapenem-resistant *A. baumannii* (Ssekatawa et al., 2018). It is important to note that factors such as improper use of antibiotics, a lack of infection control, and quick accessibility to hospitals have contributed to the occurrence of these scenarios. Older antibiotics such as colistin are a potential treatment option, although resistance to them was also emerging. *A. baumannii* accounted for 4.95% of all *Acinetobacter* species found in East Africa, while MDR, carbapenem, and pan resistance were, 87.3, 64.8, and 25.2%, respectively. Most importantly, in the present study, 77.3% of *A. baumannii* isolates were multidrug resistant.

In the present study, *Pseudomonas aeruginosa* demonstrated resistance to several commonly used antibiotics, including meropenem, ceftazidime, piperacillin–tazobactam, and ciprofloxacin, which may be attributed to multiple intrinsic and acquired resistance mechanisms. The annual resistance trend showed a temporary decline followed by a subsequent increase, indicating fluctuating susceptibility patterns over time. Clinical isolates of *P. aeruginosa* may exhibit resistance to carbapenems, aminoglycosides, cephalosporins, and quinolones through various mechanisms, including horizontal gene transfer, overexpression of efflux pumps, and production of antibiotic-inactivating enzymes. Similarly, Althaferi et al. (2025) reported that all 100 *P. aeruginosa* isolates analyzed in Kuwait exhibited elevated resistance to carbapenems and ciprofloxacin, with 33% classified as multidrug resistant. In that study, aminoglycosides showed the lowest resistance rate (31.5%). The resistance determinants identified included *blaVEB, blaVIM, aac(6’)-Ib, and qnrS* genes (Althaferi et al., 2025).

Among the most common bacterial pathogens causing hospital-acquired infections in this study was *E. coli*, particularly in ICU settings. Resistance to trimethoprim/sulfamethoxazole exceeded 70%, while ciprofloxacin resistance approached 65%. The rising trend of *E. coli* resistance to commonly used antibiotics in hospitals is a serious health issue that threatens public health. In this study, a high level of resistance was observed to trimethoprim/sulfamethoxazole and ciprofloxacin in these *E. coli* strains. In contrast, very low resistance was noted to cefixime and levofloxacin over time. These resistance patterns are largely driven by bacterial mechanisms such as enzymatic antibiotic degradation and active efflux pump systems, which reduce intracellular antibiotic concentrations and compromise therapeutic efficacy (Arbab et al., 2022). Therefore, infections become harder to treat, leading to more illness, death, and spread of disease. To combat this rising issue, additional programs focused on controlling antibiotic resistance and developing new treatment methods are urgently needed.

A major limitation of this study is the absence of routine MRSA testing using molecular detection of *mecA/mecC* genes, which limits comparison with regional and global surveillance data.

Another limitation of this study is the absence of recorded isolates of *Enterobacter* spp. and *Enterococcus faecium* in 2021 and 2022, which limited the completeness of the dataset and may have affected the accuracy of trend analysis for these organisms.

## Conclusion

The rising prevalence of MDR-ESKAPEE pathogens, especially carbapenem-resistant *A*. *baumannii* and *K*. *pneumoniae*, in various populations admitted to the ICU highlights the urgency of strengthening prevention measures, antimicrobial stewardship, routine surveillance, and infection control. AMR in Somali ICUs can be addressed by developing context-specific empiric therapy guidelines and improving diagnostic capabilities.

## Data Availability

The original contributions presented in the study are included in the article/supplementary material, further inquiries can be directed to the corresponding author.
